# Costs of the police service and mental healthcare pathways experienced by individuals with enduring mental health needs

**DOI:** 10.1192/bjp.bp.114.159129

**Published:** 2017-02

**Authors:** Margaret Heslin, Lynne Callaghan, Barbara Barrett, Susan Lea, Susan Eick, John Morgan, Mark Bolt, Graham Thornicroft, Diana Rose, Andrew Healey, Anita Patel

**Affiliations:** Margaret Heslin, PhD, King's Health Economics, Institute of Psychiatry, Psychology & Neuroscience, King's College London, London; **Lynne Callaghan**, PhD, Plymouth University Peninsula Schools of Medicine and Dentistry, Plymouth; **Barbara Barrett**, PhD, King's Health Economics, Institute of Psychiatry, Psychology & Neuroscience, King's College London, London; **Susan Lea**, PhD, Department of Psychology, Social Work & Counselling, University of Greenwich, London; **Susan Eick**, BSc, Faculty of Health and Human Sciences, Plymouth University, Plymouth; **John Morgan**, DipClinPsychol, Centre for Mental Health and Justice, Cornwall Partnership NHS Foundation Trust, Bodmin; **Mark Bolt**, PGCE, Devon & Cornwall Constabulary, Camborne, Cornwall; **Graham Thornicroft**, PhD, **Diana Rose**, PhD, **Andrew Healey**, PhD, **Anita Patel**, PhD, King's Health Economics, Institute of Psychiatry, Psychology & Neuroscience, King's College London, London, UK

## Abstract

**Background**

Substantial policy, communication and operational gaps exist between mental health services and the police for individuals with enduring mental health needs.

**Aims**

To map and cost pathways through mental health and police services, and to model the cost impact of implementing key policy recommendations.

**Method**

Within a case-linkage study, we estimated 1-year individual-level healthcare and policing costs. Using decision modelling, we then estimated the potential impact on costs of three recommended service enhancements: street triage, Mental Health Act assessments for all Section 136 detainees and outreach custody link workers.

**Results**

Under current care, average 1-year mental health and police costs were £10 812 and £4552 per individual respectively (*n* = 55). The cost per police incident was £522. Models suggested that each service enhancement would alter per incident costs by between −8% and +6%.

**Conclusions**

Recommended enhancements to care pathways only marginally increase individual-level costs.

In the UK, the importance of investment in the interface between National Health Service (NHS) mental health services and the criminal justice system has been highlighted,^1^ and research has identified substantial gaps between the sectors for individuals with enduring moderate to severe mental health needs.^2–4^ Mental health disorders are costly to society, with estimates of healthcare costs in England at around £22.5 billion per year,^5^ exclusive of indirect costs such as costs to the criminal justice system. In light of recommendations from key policy documents in recent years, this study aimed to map current care pathways between mental health services and the police, to estimate the costs to each sector and to explore, by decision modelling, the potential cost impacts of implementing enhanced care pathways based on key policy recommendations in recent years. To date, no investigation of the potential cost impacts of implementing enhanced care pathways has been conducted. The modelling approach used here is helpful in the absence of ‘harder’ evidence on the cost impacts and can prove informative for service/policy evaluation and appraisal.^5^

## Method

### Data source

Individual-level data representing current practice were obtained from the Interface study.^6^ This case-linkage study was based on data from a single rural county site in England. A random sample of cases were selected from individuals who were: recorded on the police Neighbourhood Harm Reduction Register (NHRR) or the National Strategy for Police Information Systems (NSPIS) Custody system as having interacted with the police in the second quarter of 2011; and had a record (from any time period) on the RiO electronic patient record system within the local mental care trust. Individuals were identified retrospectively and followed-up using police and mental health case records for 1 year.

### Resource use

We aimed to map current pathways through mental health and police services. This case-linkage study was novel and complex, so it was not possible to collate resource use data using standard questionnaires or data extraction tools. Instead, Microsoft Excel spreadsheets were created to systematically and iteratively record resource use information from manual reviews of individual case records. The initial spreadsheet listed a comprehensive set of services but additional services identified during the record review were appended to the spreadsheet when they first arose; use of such additional services were thus sought out in subsequent records, reviews and zero use was assumed for prior record review since the item was not documented. Recorded resource use covered a wide range of mental healthcare (including: in-patient services; client contacts with mental health staff; meetings in the absence of client; and client assessments), police and other emergency services (including: police contacts/attendance, ambulance attendance at incident), custody services (length of stay in custody suite, Mental Health Act assessments, healthcare practitioner triage, forensic medical examiner, approved mental health practitioner, hospital attendance) and other services (transport, follow-up calls by police and escorting).

### Costs of current pathways

Unit costs were attached to individual-level resource use to calculate total costs per client from mental healthcare and police service perspectives based on their recorded current pathways through these two sectors. The complexity of the study and the population necessitated including only anticipated major cost drivers in the cost analysis,^5^ to provide an estimate of the general order of magnitude of cost impacts linked to police/mental healthcare pathways. Costs covered a 1-year retrospective period for each case (thus discounting was unnecessary) and are reported in pounds sterling at 2011/2012 prices. Unit cost estimates, their sources and any assumptions made for their estimation are detailed in online Table DS1.

Costs are reported as means with standard deviations, interquartile ranges (IQRs) and 95% confidence intervals (obtained by non-parametric bootstrap regressions; 1000 repetitions; bias corrected and accelerated). We explored predictors of costs by univariate bootstrap regressions (ordinary least squares) of costs and the following baseline characteristics: age; gender; marital status; living situation; ethnicity; employment status; referral status (substantive (on the case-load of a care team for more than 2 months at the time of the index police contact) *v.* non-substantive (on the case-load of a care team for less than 2 months at the time of the index police contact)); and residence (out of county *v.* in county). Data were analysed using Stata version 11.

### Modelling enhancements to current care pathways

Decision models are a methodical way of thinking about the likely impact of a decision (for example to add an enhancement to a care pathway or not). They involve constructing a model of the core elements of current practice, and then making alterations to this model to represent alternative care pathways. They can include inputs (costs) and outputs (outcomes) associated with each care pathway to inform resource allocation decisions under conditions of uncertainty.^7^ To explore the potential impact of enhancing current care pathways through the implementation of recommendations in key policy documents, we first developed a decision model that represented current mental health and police costs per incident using the case-linked data (the base case). In a decision model, the proportion of clients following specific cost-generating pathways subsequent to key decision points in the system is represented by probability values. Probability values were calculated from the case-linked data based on all incidents (55 clients with a total of 783 incidents; Table 1). Models were built using Microsoft Excel and their structures were validated by relevant stakeholders (mental health professionals and senior police officers).

**Table 1:** Probabilities of events for the decision modelling^a^

Event point	*P*
Following police attendance:	
Section 136 detention	0.03
Arrest	0.18
No further action	0.80

*Section 136 detention*	
Following Section 136 detention:	
Take to Section 136 suite	0.14
Take to custody	0.86
Following Mental Health Act assessment following being taken to Section 136 suite:	
Detained – transfer to hospital	0.00
Not detainable – release	1.00
Following being taken to custody:	
Forensic medical examiner assessment	0.08
Healthcare practitioner assessment	0.83
No assessment	0.08
Following forensic medical examiner assessment:	
Mental Health Act assessment	0.00
Not detainable – release	1.00
Following healthcare practitioner assessment:	
Forensic medical examiner assessment	0.70
Mental Health Act assessment	0.30
Not detainable – release	0.00
Following forensic medical examiner assessment after healthcare practitioner assessment:	
Mental Health Act assessment	0.71
Not detainable – release	0.29
Following Mental Health Act assessment after forensic medical examiner assessment following healthcare practitioner assessment:	
Detained – transfer to hospital	0.40
Not detainable – release	0.60

*Arrest*	
Following Mental Health Act assessment after forensic medical examiner assessment following healthcare practitioner assessment:	
Forensic medical examiner assessment	0.01
Healthcare practitioner assessment	0.67
No assessment	0.31
Following forensic medical examiner assessment:	
Mental Health Act assessment	1.00
Not detainable – release	0.00
Following healthcare practitioner assessment:	
Forensic medical examiner assessment	0.14
Mental Health Act assessment	0.86
Not detainable – release	0.00
Following forensic medical examiner assessment after healthcare practitioner assessment:	
Mental Health Act assessment	0.25
Not detainable – release	0.75
Following Mental Health Act assessment after forensic medical examiner assessment following healthcare practitioner assessment:	
Detained – transfer to hospital	0.50
Not detainable – release	0.50

a. Some probabilities equal >1 due to rounding. Source of information: clients with a police attendance in the interface case-linkage study.

By necessity, models are simplified representations of actual care pathways. We therefore included only key services whose costs were expected to contribute the greatest to total mental healthcare and police costs: police attendance, custody, assessment and mental health in-patient care. These services represent 82.5% of total mental health and police costs in the case-linked data. In-patient services have previously been found to be the major cost driver for mental health service costs.^8^

**Fig. 1:**
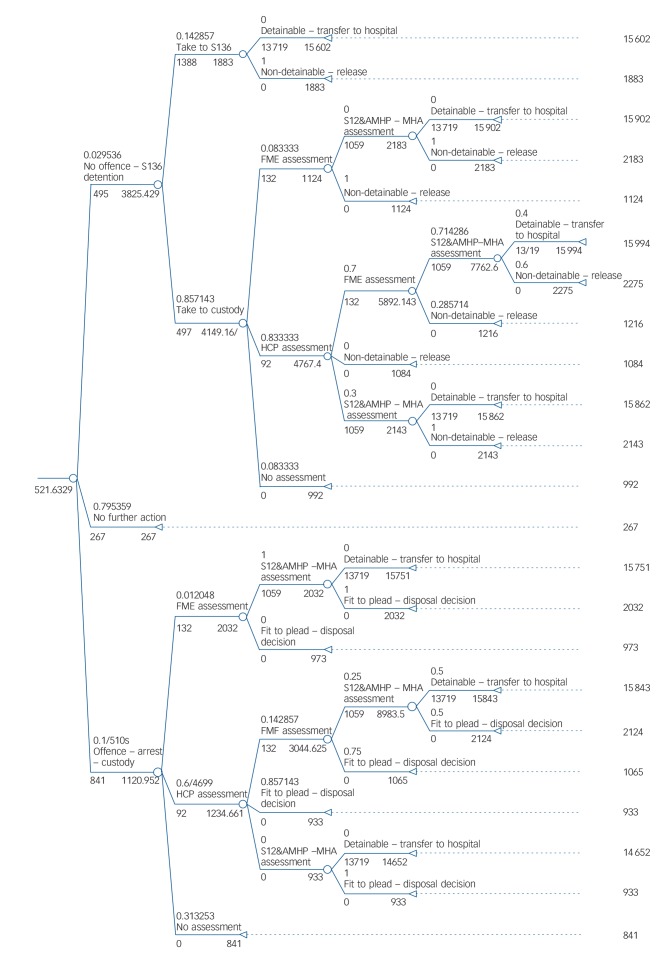
Model 1: current care pathways (base case). S136, Section 136; S12, Section 12; AMHP, approved mental health practitioner; MHA, Mental Health Act; FME, forensic medical examiner; HCP, healthcare practitioner.

After estimating total mental healthcare and police costs within the structure of simplified current care pathways (the base case; Fig. 1), we explored the potential impact on costs of implementing three recommendations from key policy documents: street triage; Mental Health Act assessments for all individuals detained under the Mental Health Act Section 136 (i.e. individuals taken from a public place to a hospital for a psychiatric assessment); and a link worker at custody suites. Street triage and custody liaison interventions are the two main government-funded initiatives to come out of the Bradley Report.^3^ The custody liaison service in the study site commenced just as the research window ended and there is still no formal street triage service in this area. Mental Health Act assessments were not mandatory in the study site at the time of the study and there was local concern that there would not be sufficient resources to implement such an initiative.

Street triage involves mental health nurses accompanying officers to incidents where there is an indication that someone is in need of mental health support.^9^ Its role is to provide assessment, care and treatment as quickly as possible. Initial pilots show that street triage can help keep people out of custodial settings thus reducing demands on police time. This relates to the recommendations that all custody suites should have a liaison and diversion officer.^3^ Effectively, street triage is a liaison and diversion officer who is based in the field, rather than in custody suites and has the added advantage of diverting unnecessary Section 136 detentions, rather than just diverting service users once a Section 136 has been implemented. It was assumed that implementing street triage would increase the cost of each police attendance by £53 per hour of client contact (assuming the same cost as assertive outreach; online Table DS1) and that it would have no effect on the number of incidents in which the police took no further action or that resulted in the arrest of the client.

Preliminary findings from the Cleveland Mental Health street triage pilot were that of the 371 people assessed by street triage, only 3.2% of them needed to be detained for a Section 136 assessment. We thus conservatively estimated that the probability of going to a Section 136 suite or custody for Section 136 assessment would be reduced by 50%, and that half of clients entering through that arm of the pathway would avoid being put under a Section 136 and be referred to appropriate services via street triage contacts. These assumptions are based on an informed judgement in the absence of evidence of the impact on policing and other criminal justice activity.

The case linkage data indicated that not all Section 136 detainees were receiving a Mental Health Act assessment despite the Mental Health Act 1983 statement that all detainees under Section 136 should receive assessment by a medical practitioner and an approved mental health professional. We therefore explored the impact of implementing Mental Health Act assessments for all Section 136 detainees. This removes the need for forensic medical examiner and healthcare practitioner assessment and has an impact on the likelihood of detention.

The service enhancement of a link worker at custody suites is developed from a number of documents, most notably the Bradley Report,^3^ which states that ‘all police custody suites should have access to liaison and diversion services’. We modelled the introduction of a link worker covering clients in custody on an arrest (as this is what is currently being implemented in the site where data were collected). We therefore added the cost of a link worker (see online Table DS1). Probabilities within the model remained the same as in the base case.

**Fig. 2:**
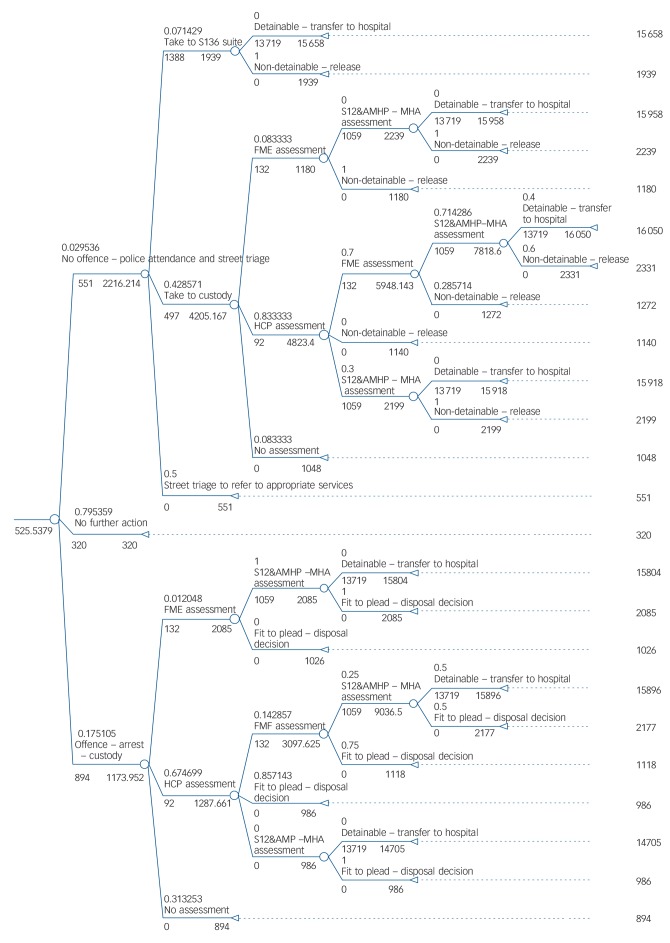
Model 2: current care pathway enhanced by the addition of street triage. S136, Section 136; S12, Section 12; AMHP, approved mental health practitioner; MHA, Mental Health Act; FME, forensic medical examiner; HCP, healthcare practitioner.

**Fig. 3:**
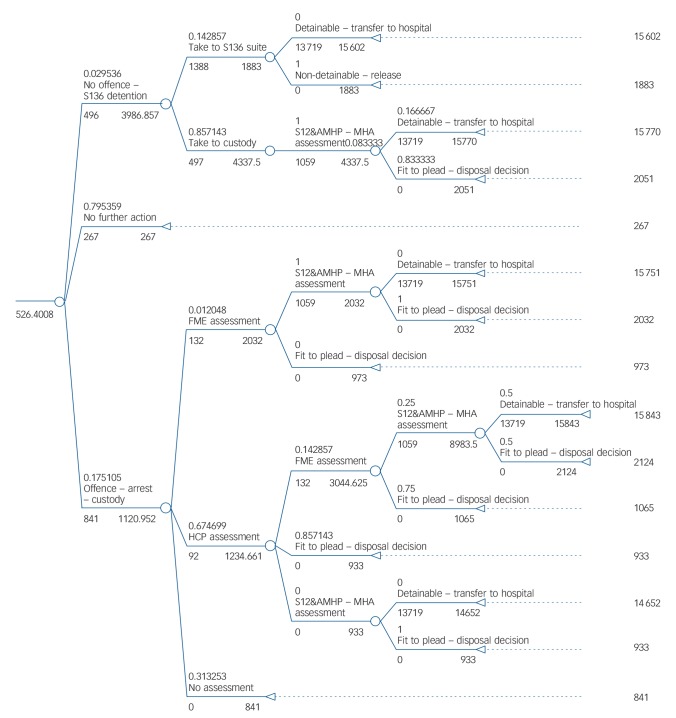
Model 3: current care pathway enhanced by Mental Health Act assessment for all Section 136 detainees. S136, Section 136; S12, Section 12; AMHP, approved mental health practitioner; MHA, Mental Health Act; FME, forensic medical examiner; HCP, healthcare practitioner.

**Fig. 4:**
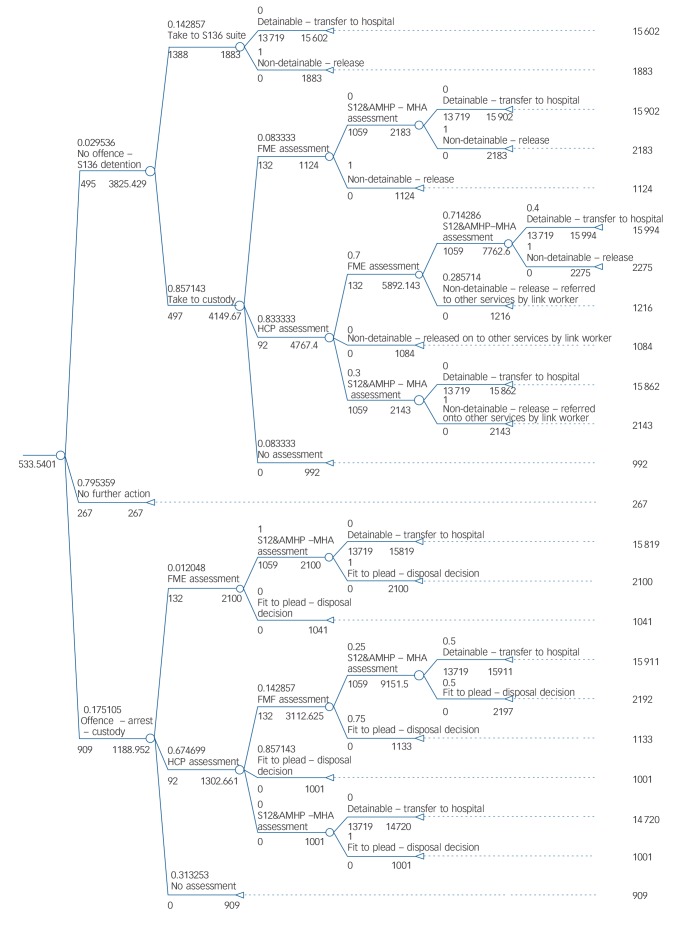
Model 4: current care pathway enhanced by link worker at custody level. S136, Section 136; S12, Section 12; AMHP, approved mental health practitioner; MHA, Mental Health Act; FME, forensic medical examiner; HCP, healthcare practitioner.

Modelling pathways incorporating these three enhancements involved generating alternative structures and/or alternative probability values based on assumed deviations from probabilities observed in the case-linked data. These adjustments were informed by policy recommendations or subsequent changes to practice in the site where data were collected that have since been adopted or are currently being advised (see Figs 2, 3 and 4 for details). One-way deterministic sensitivity analyses were used to assess the sensitivity of conclusions to the assumptions made. These are detailed within the Results section alongside the associated findings for ease of understanding.

### Ethics

Ethical approval was granted from the relevant NHS research ethics committee, trust research and development office and ethics committees within the Higher Education partners and section 251 of the NHS Act was granted setting aside the common law duty of confidentiality to access records without consent for the purpose of research.^10^

## Results

### Resource use and costs

Resource use data were collected for a random sample of 55 out of the 80 individuals in the case linkage study. All individuals had at least one contact with mental health services during the 1-year period following the index contact with the police. Thirteen individuals (24%) had at least one in-patient stay with a mean of 90 in-patient days (s.d. = 92, range 5–310 days). Forty-nine individuals (89%) had at least one client assessment, 52 (95%) had at least one meeting with a professional and 37 (67%) had at least one meeting between professionals in the absence of the client. The mean mental healthcare cost per person over the 1-year period was £10 812 (s.d. = £23 714; IQR = £386–£6335, 95% CI £6055–£19 726). In-patient services accounted for 76% of total mental healthcare costs. The next largest cost drivers were meetings with professional staff (13%), client assessment (9%) and professional meetings in the absence of the client (2%).

All individuals had a recorded contact with police as a result of the sampling strategy. The median number of contacts (separate incidents) with the police was 7 (IQR = 3–14). One individual was an outlier with 293 contacts. The total number of police contacts for the whole sample was 783; 461 (59%) of these required at least one police officer attendance. Of those incidents with a police officer in attendance, the median number of officers was 3 (IQR = 2–5). Of the 783 police contacts, 98 (13%) involved some time in custody, of which the median time in custody was 8 h 38 min (IQR = 4 h–15 h 30 min). In total 12% (12/98) of custody contacts had a Mental Health Act assessment. The mean police service cost per person was £4552 (s.d. = £4461, IQR = £1360–£7020, 95% CI £3551–£6058). The major cost driver for police costs was police attendance at incidents, accounting for 59% of total costs (online Fig. DS1). This was followed by custody costs.

The mean total mental health and police cost per person was £15 364 (s.d. = £24 007, IQR = £2647–£14 962, 95% CI £10 689–£24 960). The only baseline characteristic associated with costs was whether the client was a substantive referral or not, with non-substantive referrals on average costing an additional £12 850 (95% CI £3945–£29 192).

### Costs of implementing service enhancements

#### Street triage

Current pathways through care incurred average costs of £522 per incident (Fig. 1). Street triage increased the average cost per incident from £522 to £526 (i.e. by less than 1%). Reducing the probability of a client entering the street triage referral arm to 20% increased per incident costs to £555 (+6%). A further sensitivity analysis assumed street triage only influenced entry into custody via a Section 136, and not through entry into Section 136 suite. This reduced those taken to custody on a Section 136 by 20%. Based on these assumptions, the average cost per incident was increased to £556 (+6%). If the preliminary finding that only 3.2% of individuals need to be detained for Section 136 assessment is assumed to be representative, and the other 96.8% can avoid Section 136 detention and be referred to appropriate services by street triage, the average cost per incident was decreased to £478 (−8%). A further sensitivity analysis was conducted based on the finding that of those put on a Section 136, only 25% end in a detention, with the other 75% progressing through the pathway without ending in a detention. If street triage were to prevent the 75% who do not end in detention from entering the Section 136 arm, the average cost per incident would be £501 compared with £522 in the current pathway (−4%). Although in each of these analyses street triage is assumed to reduce either the number of individuals entering custody on a Section 136 or the number of individuals entering custody on a Section 136 plus the number of individuals being taken to Section 136 suites, the resulting cost reductions do not offset the added cost of street triage but remain modest.

#### Mental Health Act assessments for all Section 136 detainees

This enhancement similarly increased costs by less than 1% (from £522 per incident to £526). Since practice at the study site now includes contact with a forensic medical examiner in all custody cases as well as a Mental Health Act assessment, we also explored the cost impact of this and found average costs increased to £530 per incident (+2%). The more intensive input scenario of a forensic medical examiner contact and healthcare practitioner in all custody cases in addition to the Mental Health Act assessment led to a similar cost increase (to £532; +2%).

#### Link worker at custody suites

This enhancement increased costs to £534 (+2%). A sensitivity analysis that assumed a client contact duration of 3 h rather than 1 h increased per incident costs to £557 (+6%).

## Discussion

Individuals on the case-load of a care team for less than 2 months at the time of the index police contact had higher costs compared with individuals who had been on the case-load for at least 2 months. This is an important finding that offers an area of focus for service developments aiming for cost containment. Similarly, we identified in-patient services and police attendance at incidents as the major cost contributors and this gives some insight into the potential benefits to police and the healthcare system if enhanced pathways could effectively manage and improve behavioural outcomes in this client group.

Our models of the potential impact of introducing enhancements to care pathways suggested minimal effects on individual-level average costs, which held under various sensitivity analyses. Although it could be argued that small increases in individual-level costs could have substantial budgetary implications when such enhancements are scaled up, there may be parallel improvements in client/societal outcomes that justify additional expenditure (‘intervention at the police station may help prevent more serious offending’^10^). Unfortunately, it was beyond the scope of this study to model such outcomes and any associated knock-on or long-term cost savings.

### Strengths and limitations

Decision models carry some general limitations. For example, many probabilities in the enhanced pathway models are based on informed assumptions in the absence of hard evidence. We attempted to mitigate the impact of this by seeking external validation of the model structure and assumptions from relevant stakeholders. Further, simplification of care pathways necessarily carries limitations. For example, our examination of Mental Health Act assessments does not account for the knock-on effect of increased time in custody because of increased waiting time for these assessments, which stakeholders informed us was likely if more clients require an assessment. The complexity of the data linkage approach necessitated a focus on police and mental health service costs. Cost findings may not represent pathways in other areas given the considerable national variation in how people are managed in the interaction between police and wider services. A further limitation is the exclusion of people who have mental health needs that are being met by primary care, or not being met at all. These individuals may be harder to reach and thus the current economic modelling would not fit their service patterns. Although examining the cases of individuals not known to mental health services was beyond the scope of this study, this is incredibly important for future work in this area.

The study also has a number of strengths. First, our estimates of current care costs and probabilities were based on observed activity (albeit for a limited number of individual cases) linked to existing pathways. This formed the basis of our models of the potential costs of enhanced pathways and provided a robust base case against which they could be compared. Second, we avoided overly complex and, therefore, non-transparent, models and have focused on identifying and estimating key cost drivers linked to the identified service enhancements.

### Implications for future research

Given the focus of our case linkage study, future evaluations of the interplay between police and other services could additionally explore the use and costs of physical healthcare and specialised accommodation by this client group, which could contribute substantial further costs to our estimates. Most important, however, is the need to consider client and wider societal benefits as assessments of costs alone do not inform decisions about whether service enhancements offer value for money.
